# Autologous fat transfer with *in-situ* mediation (AIM): a novel and compliant method of adult mesenchymal stem cell therapy

**DOI:** 10.1186/1479-5876-11-136

**Published:** 2013-05-31

**Authors:** Allan Y Wu, David M Morrow

**Affiliations:** 1 The Morrow Institute, 69-780 Stellar Drive, Rancho Mirage, CA 92270, USA

## Abstract

**Background:**

In an attempt to engineer a regulatory compliant form of cell assisted lipotransfer in the U.S., the authors developed Autologous Fat Transfer with *In-situ* Mediation (AIM) for reconstruction of a refractory surgical scar.

**Methods:**

This method incorporates use of accepted standard procedures like autologous fat grafting and intradermal injection of NB6 collagenase to release adipose stem cells from a naturally occurring high concentration stromal vascular fraction (SVF) fat graft. To prevent off-target effects of collagenase, a hyaluronic acid and serum deactivation barrier is placed circumferentially around the operative site.

**Findings:**

This novel protocol was well tolerated by the patient and improved scar appearance, mobility and texture. Deepest scar contour defect correction was 80% and 77% at 4 and 12 weeks respectively.

**Conclusion:**

AIM appears to be a practical and viable option for scar reconstruction requiring small to moderate volume correction.

## Background

Cell assisted lipotransfer (CAL), a process whereby free fat grafting is enriched with a supraphysiologic level of autologous stromal vascular fraction (SVF) cells (which include autologous adipose stem cells), has been utilized for nearly a decade in Japan for tissue augmentation and reconstruction [1]. Thus far CAL, under appropriate conditions, appears to be clinically efficacious and safe [2]. Within the United States, practitioners are confronted with regulatory issues of 21 CFR 1271 and concerns that a lengthy Investigational New Drug (IND) process is necessary for CAL use to be legal according to formal Requests for Determination now disclosed in the public domain. Further questions remain as to whether or not a key step in CAL (*ex-vivo* enzymatic separation of SVF and concentration by centrifugation) constitutes a greater than “minimal manipulation” procedure despite being done in the same surgical setting. The defining lower limits of what constitutes “minimal manipulation” have not been fully articulated directly by the Food and Drug Administration (FDA) at this juncture, and for this important reason plastic, cosmetic and reconstructive surgeons within the States struggle to find a practical and clearly compliant means of providing some form of CAL or adult mesenchymal stem cell therapy. Meanwhile the ever marching reality of refractory wounds, scar revisions, congenital abnormalities which have exhausted standard medical therapy continue to grow and remain untreated.

Historically, the procedures of: 1. autologous fat transplant (AFT), 2. *in-vivo* high quality current good manufacturing practice (cGMP) grade collagenase injection, 3. dermal chemical peeling and 4. dermal hyaluronic acid (HA) fillers are widely considered acceptable, safe and routine standards of medical care in the United States and abroad. We postulated on the outset of this case study that fat retaining pre-existing high concentrations of SVF/ADSC could be transferred under routine AFT methods, but further treated with collagenase subcutaneously to release or migrate ADSC off the collagen matrix into a wound bed or chemically peeled skin. A “ring” or moat of HA admixed with autologous serum surrounding the collagenase could also be placed as a deactivation barrier to prevent off-target or off-site effects of collagenase. This “under the skin” mediated approach using accepted standard of care techniques and materials avoids contentious *ex-vivo* manipulation, thereby allowing practitioners a potentially regulatory compliant form of regenerative surgery for soft tissue reconstruction. We have combined the stated standard of care procedures in a unique sequence to treat a patient that failed standard fat transfer for correction of a contracted and excavated scar sustained after previous lipoma excision.

## Methods

Patient was selected for having failed previous standard AFT 2 years previously for correction of an adherent cicatrix scar sustained on the right lower back after lipoma excision. The procedure was performed in a fully accredited ambulatory surgery center staffed by a physician anesthesiologist. A comprehensive and thorough pre-operative consultation and specific consent for percutaneous aponeurotic lipofilling (PALF) with AFT in conjunction with collagenase and HA-serum was obtained.

**Figure 1:**
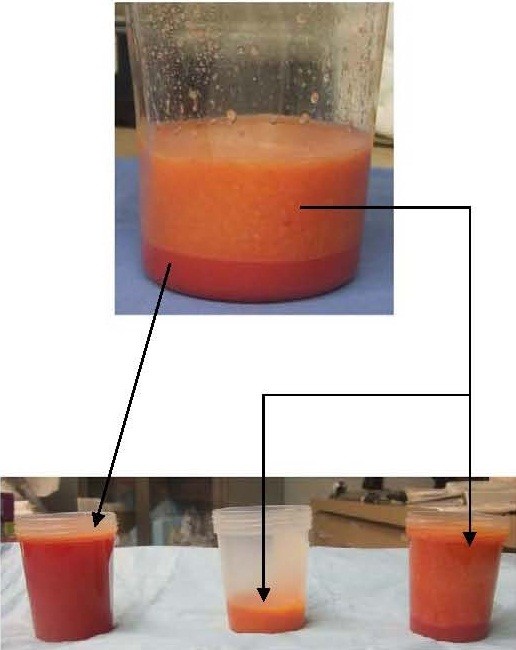
**Images of standard lipoaspirate (top container) after Coleman technique separated into aqueous phase (far left container) and yellow appearing low SVF fat (center container) and orange appearing high SVF (right container).** (Oil phase not pictured.) Because the fat is already pre-washed, the heme pigmentation is not a result of contaminated red blood cells (RBCs), but rather RBCs that are still within the perivascular space thereby creating an indirect marker for a fat fraction rich in perivascular cells (i.e. orange appearing fat).

**Figure 2:**
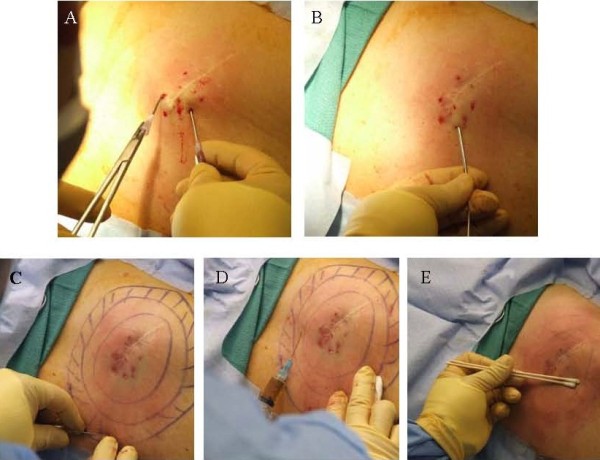
**Images of procedures utilized for AIM technique.** PALF maneuvers (**A**) are done prior to autologous fat grafting (**B**). A concentric ring and undermined cross-hatching with an HA-serum barrier (**C**) is placed prior to collagenase NB6 injection (**D**). (Note the natural amber appearance of collagenase). Overlying skin, but not scar, is then chemically treated with 10% TCA in a single “wet” coat without deep scrubbing and just to the point of erythema and no frost (**E**).

**Figure 3:**
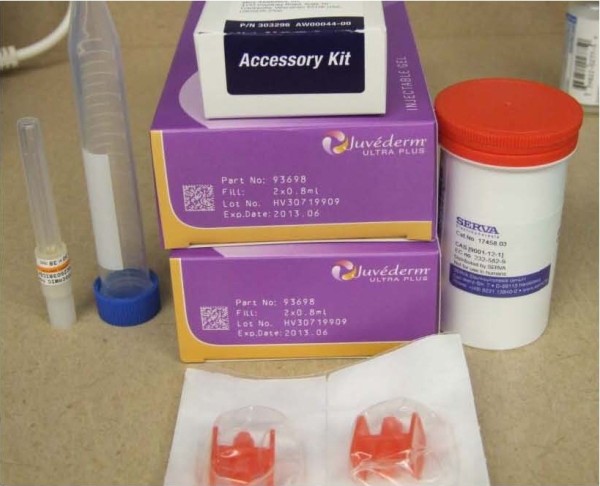
**Photograph of all FDA approved and cGMP materials used for the AIM technique.** Standard HA without lidocaine and cGMP NB6 collagenase are critical starting reagents. The blunt cannula (far left) may be substituted with a spinal needle to allow longer tracts for barrier formation. A yellow top vacutainer may also be used with 1 cc of dilute Heparin instead of a conical centrifuge tube. Red Luer lock connectors (center bottom) can be obtained in premade dermal accessory kits (center top).

**Figure 4:**
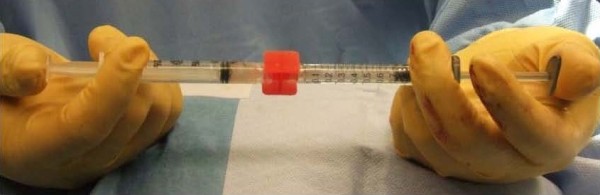
**Mixture of platelet poor serum and HA dermal filler using Luer lock connector and back-and-forth mixing with syringe pistons.** Note: the meniscus of serum and HA is brought to the highest level of the Luer lock or connector hub prior to mixing to minimize presence of air bubbles.

Autologous fat was harvested from the anterior abdominal wall using standard Klein tumescent solution and a 2 mm inner diameter cannula (Mentor BENSAT 330). Negative pressure was limited to 350 mmHg. Lipoaspirate was washed three times with normal saline and gravitational decanting was used to remove as much aqueous phase possible. Fat was then centrifuged and purified under standard Coleman technique (i.e. centrifugation at 1,000 RCF for 3 minutes followed by immediate decantation of oil and aqueous phase) [3,4]. Lipoaspirate was further processed to separate SVF rich fat (Figure 1) using a non-invasive proprietary method of spectroscopy (Patent publication number EP2346989 A1). PALF was performed on the subdermal bed of the scar directly adherent to the erectus spinae muscle (Figure 2A) [5]. AFT was performed by the minimal barotrauma method of reverse injection at 3-5 cc per pass layering thin ribbons of graft using a blunt spatulated tip (Marina Medical 20–1415) through 6 strategically placed 5 mm adits at 20% overcorrection at the deepest excavation point using 100 cc total (Figure 2B). A 2 cm wide ring of SVF rich fat (60 cc) was distributed circumferentially around the wound bed. A perimeter of and underlying layer of HA-serum filler (0.5 cc autologous serum to 1 cc of HA filler) was placed in a cross-hatched fashion using a 22 gauge spinal needle (Figures 2C, 3, and 4).

Collagenase was prepared by serially diluting NB6 collagenase (GMP grade 17458 SERVA Electrophoresis, Nordmark GmBH, Crescent Chemical) with PBS (GMP grade D8662 Sigma) to a final concentration of 0.1 PZ-U/mL (Figures 2D and 3). A total of 10 cc of dilute NB6 was distributed with a 25 gauge needle within the dermis holding SVF rich graft. Wet application of T10 chemical peel, without deep scrubbing, was placed over the skin directly overlying the PALF wound bed, but not directly above the scar itself. After the skin was left to air dry a non-adherent (100540/960482, Kendall, Tyco Healthcare) occlusive dressing (1626, 3 M Corporation) was applied. Patient was placed on activity restriction to prevent direct compression of the nascent graft and received 3 days of post operative IV antibiotics (Cefotetan 2 gm q24 hours) followed by 7 days of oral antibiotics (Keflex 500 mg BID).

Post operative photography and ultrasounds (Acuson 7 mHz flat probe) were obtained days 1–5 and 1, 2, 4, 8 and 12 weeks following surgery.

## Results

PALF and AFT were able to immediately resolve adherence of the scar directly against the muscle without need or use of complete scar subcision. Manual palpation post-operative days 1 through 5 and weeks 2 through 4 did not reveal any subcutaneous fremitus or hypermobility of scar.

**Figure 5:**
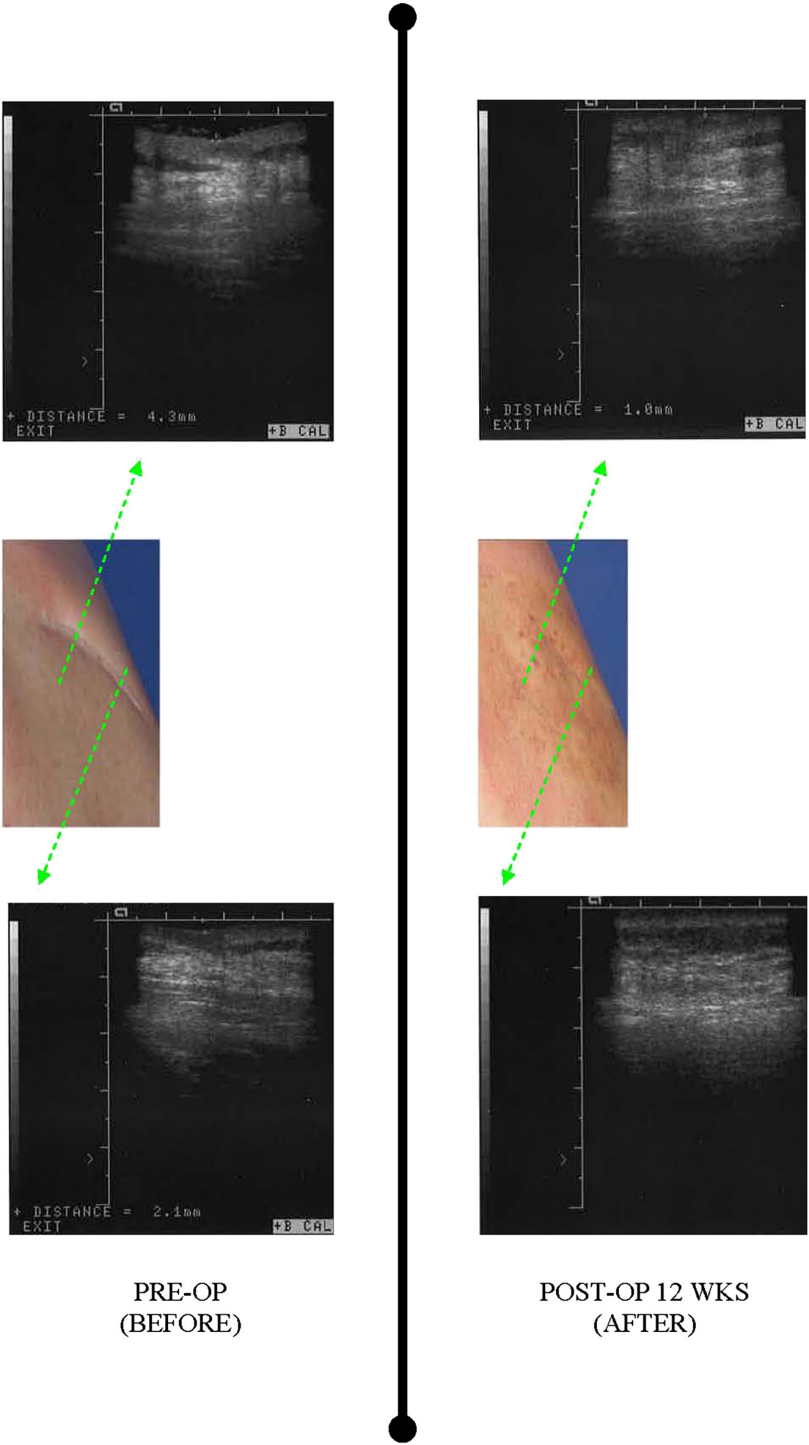
**Before (left panel) and after (right panel) photographic images and corresponding ultrasounds (green arrows) documenting correction of contour defect seen at 12 weeks post procedure.** Ultrasound images were obtained using a 7 mHz probe and placed perpendicular to the long axis of the scar to obtain reproducible cross-section views of excavated dermis.

**Figure 6:**
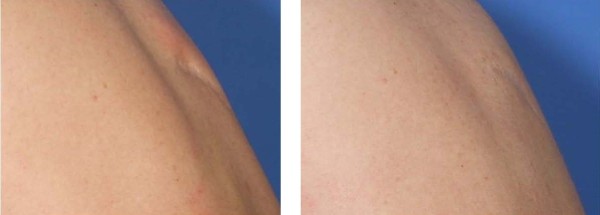
**Before (left panel) and after (right panel) photographs of scar during full flexion of back accentuating medial excavation or “atrophy” of subcutaneous fat and upper dermis tissue (left panel).** Post operative image of same scar following AIM procedure with back in full flexion showing sustained augmentation and engraftment to relatively avascular scar tissue bed. Note more smooth appearing contour.

**Figure 7:**
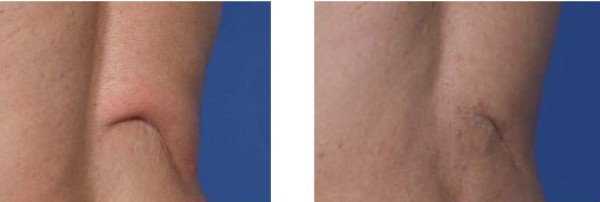
**Before (left panel) and after (right panel) photographs of scar during full flexion of right erectus spinae muscle showing softening and remodeling of scar and overlying skin at 12 weeks post procedure (right panel) in addition to less movement and adherence of scar with muscle flexion.** Note that both erectus spinae muscles of the back were fully functional as local lidocaine was fully dissipated and metabolized at 12 weeks and ultrasound was still able to confirm the underlying voluntary movement and flexion of muscles (not pictured).

Unlike typical AFT, the wound bed did not exhibit the typical induration swelling phase, and echymossis formation was minimal. An unexpected finding at 12 hours post procedure was the complete epithelialization of the six operative incisions (5 mm) over the wound bed. In comparison, the 5 mm incisions over the abdomen used for liposuction were not even partially epithelialized despite absence of drainage and placement of 3–0 Nylon sutures immediately following liposuction. Desquamation of overlying skin was not appreciated with the chemical peel. Qualitatively the lateral most aspects of the scar exhibited signs of remodeling based on softening texture, fibrotic scar height and color blending with surrounding normal skin (Figures 5, 6 and 7). Pre-operative pain associated with extreme flexion of the right erectus spinae muscle resolved at 6 weeks.

Ultrasound revealed correction of 77% of the original excavated defect at the maximal depth. The lateral aspect of the scar showed 100% correction at 3 months out from the original procedure (Figure 5).

## Discussion

The authors emphasize that based on current literature CAL appears to be a safe and efficacious procedure [6-8]. Although CAL may be performed under an IRB or IND based clinical study in the U.S., the reality of not being able to perform procedures unapproved by the FDA, either from a liability insurance or institutional policy standpoint, precludes many a North American practitioner from providing the service. Trichloroacetic acid (TCA) chemical peeling of the skin has been widely used through out dermatology and plastic aesthetic surgery for skin resurfacing and remodeling [9,10]. Collagenase has been utilized *in-vivo* extensively in reconstructive surgery to facilitate anesthesia and dissection of skin flaps, fibrotic tissue and correction of misplaced dermal fillers [11-13]. It has also been commonly used as a debriding topical agent for wound care [14]. FDA approved hyaluronic acid based dermal fillers have also been safely employed for reconstructive reasons [15-17]. However, of all the procedures mentioned, autologous fat grafting has the longest track record of use well prior to the advent of modern medicine [18]. These safe and FDA allowable procedures when combined using the protocol presented, should provide some practitioners a means of practicing a form of regenerative surgery without legal regulatory repercussion. However, the use of an FDA approved material for a new indication is considered “off-label” use, and while not illegal, it is not without clinical risk if sound medical judgment is ignored. The authors also emphasize the important distinction that although the independent incremental procedures used are standard of care, many accepted standard surgical procedures performed in the United States are never granted “approval” by the FDA, as the jurisdiction of purely surgical procedures is in large part the domain of State medical boards and professional organizations. (The authors in no way state or imply that the AIM procedure is endorsed or approved by the FDA.)

Unique to this procedure is the isolation of native high SVF fat. This can be achieved by visual inspection and volumetric location alone [19]. However, with small volume fat extraction, use of non-invasive spectroscopy to measure surrogate markers of pericyte loci provides maximum harvest of this essential graft, which visual inspection can under calculate [20]. It is also important to emphasize that well characterized high grade cGMP collagenase near absent of endotoxin is critical for direct *in-vivo* use and may be prove critical for creating a directional migration of ADSC when employing this technique. Our choice of NB6 was prompted by historical data and full disclosure readily provided by the manufacturer regarding cGMP and endotoxin status (<< 11 I.U./mg). Custom compounded collagenase commercially available within the United States does not always carry as copious of manufacturing documentation, and with recent concerns of sterility and safety of compounded preparations, use of high quality cGMP material is strongly recommended for this unique application [21].

**Figure 8:**
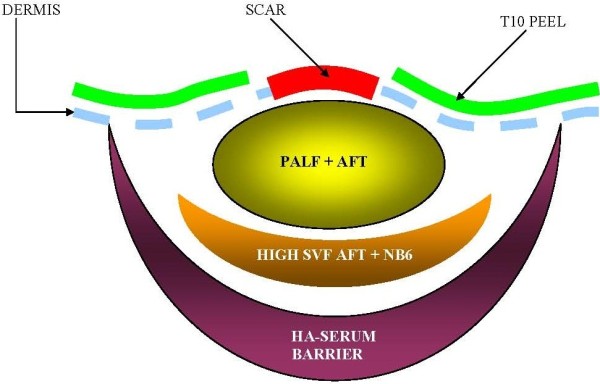
**Conceptual cross-sectional drawing of different therapeutic layers and their relationship to the scar (in red), dermis (dotted blue line) and grafts (yellow ovoid and orange crescent).** The upper fat graft (yellow ovoid) serves and the primary reconstructive and augmenting element, whereas the high-SVF graft (orange crescent) provides a rich source of ADSC/SVF cells not previously available to the wound site.

**Figure 9:**
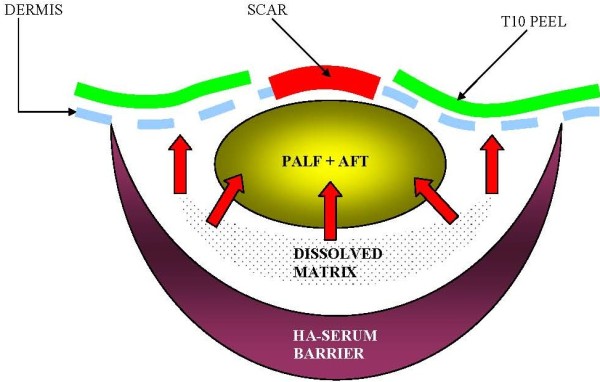
**Conceptual and theoretical cross-sectional drawing of AIM procedure after high SVF fat graft matrix is digested *in-vivo *(shaded crescent).** Adipose mesenchymal stem cells are then freed from the native fat matrix to potentially migrate in direction of wound bed or become activated to secrete paracrine mediators of wound healing and regeneration as depicted by red arrows. Note location of 10% TCA peel (green line) is NOT placed directly over the scar itself.

Due to previous keloid formation on her back, the patient refused to consent for biopsy of the wound area post operatively. This clinical dilemma has lead to a confusion of sorts with respect to precisely elucidating the exact mechanism of action for this procedure and why it was successful versus standard AFT. Mechanical dissociation and graft migration as the key mechanism is unlikely, as one would expect the majority of volume augmentation to settle towards the dependent areas of the back, which is not supported by photographic and ultrasound data. TCA causing direct scar remodeling is also another possible mechanism, but is highly unlikely given the strength of TCA used in this study is over a thousand-fold less than those used in direct scar remodeling studies [10]. Furthermore, TCA was not applied directly to the scar itself and desquamation, a common sign of deep dermal peeling, was never observed in this case. Remaining possible mechanisms include direct factor signaling [22] of detached SVF/ADSC versus migration of ADSC into the wound site from the surrounding SVF rich graft. Collagenase in this context could not only form the basis for ADSC release, but also provide microscopic conduits similar to the PALF procedure, wherein migrating cells gain direct physical pathways to areas requiring regeneration (Figures 8 and 9) [23]. Of the two remaining theories the concept of stem cell *in-vivo* migration has been suggested previously, but only from the perspective of mobilizing marrow mesenchymal stem cells [24]. The concept of *in-vivo* migration in our model system implies a lesser distance to travel and perhaps a greater local effect, since adipose tissue inherently retains one of the highest concentrations of mesenchymal stem cells for any tissue in the human body [25]. We propose AFT with *in-situ* mediation (AIM) of ADSC via light concentration collagenase *in-vivo* is a tenable form of regenerative therapy with minimal side-effect to the patient.

Although it was not the intent of this pilot study to elucidate the theoretical underpinnings for why the novel procedure works, further confirmatory studies using animal models will need to be performed. (However, cursory results of nude-mice studies, simultaneously being pursued by our group appear promising.) The authors fully acknowledge this is a single case study with remarkable results the reproducibility of which will need to be verified with further study. In light of this, three other patients have been treated using the same protocol in other regions of the body with no adverse or safety events. At this juncture, precaution is advised in extrapolating this procedure to large volume augmentations used in full breast or buttock augmentation. Use of the procedure on smaller lesions such as chronic lower extremity wounds, which allow adequate circumscribing with an HA-serum deactivation zone, is ideal and will be the focus of pending clinical investigations at our institution.

## Conclusion

*In-vivo* use of NB6 collagenase subdermally is well tolerated and well suited for the AIM technique. The AIM procedure itself also appears safe and capable of providing contour correction and soft-tissue reconstruction and augmentation where standard AFT might fail.

## Competing interest

The authors declare that they have no competing interests.

## Authors’ contribution

AYW and DMM contributed to the study design and development of surgical techniques. AYW performed diagnostic studies, medical material preparation and therapeutic procedures. Both authors read and approved the final manuscript.
