# Erratum: Autologous fat transfer with *in-situ* mediation (AIM): a novel and compliant method of adult mesenchymal stem cell therapy

**DOI:** 10.1186/s12967-014-0269-z

**Published:** 2014-09-30

**Authors:** Francesco M Marincola

**Affiliations:** Sidra Medical and Research Center, Doha, Quatar

After publication of this article [1] it was brought to the journal’s attention that David Morrow was unaware of the submission of this article in his name and did not consent to become an author. He points out that he was not involved in the creation of the article. Readers are also alerted that, in the absence of any evidence from an institutional investigation to confirm or refute the reliability of the data, there are ongoing concerns and this article’s findings should be interpreted with caution. Appropriate editorial action will be taken if further information becomes available.
