# Expression of concern: Autologous fat transfer with *in-situ* mediation (AIM): a novel and compliant method of adult mesenchymal stem cell therapy

**DOI:** 10.1186/s12967-014-0298-7

**Published:** 2014-11-04

**Authors:** Francesco M Marincola

**Affiliations:** Sidra Medical and Research Center, Doha, Quatar

After publication of this article [1], it was brought to the journal’s attention that there were several potential concerns regarding the reliability of the data presented within it.

There is uncertainty regarding the timing of the images in figures 5 and 7.

Despite extensive efforts to obtain an independent investigation, we have been unable to verify these claims or come to a definitive conclusion regarding the reliability of the data. Readers are therefore urged to take caution when interpreting the content of this article.

Readers are alerted that an erratum has also been published regarding the authorship of this article [2].
